# Improving the fundamental understanding of batteries via *operando* measurements

**DOI:** 10.1038/s41467-022-32245-9

**Published:** 2022-08-15

## Abstract

Recent development of analytical techniques – capable of characterizing physicochemical properties during electrochemical measurements – enables new pathways for improving the fundamental understanding of battery systems. This editorial highlights recent research efforts showcasing operando approaches published in Nature Communications.

Understanding the electrochemical energy storage behavior of a material is strictly tied to the framework of operation. For instance, in cases where gas adsorption/desorption characterization of an electrochemical active material is carried out at the powder level, the same properties (e.g., available specific surface area) may not be retained after the material is processed for electrode formulation. Similarly, the bulk ionic conductivity of an electrolyte solution is not representative of when the same electrolyte is confined within a porous electrode structure^1,2^. Thus, limitations arise, and what can be claimed via indirect correlation within a specific framework may not be meaningful when the same material is constrained within a different system.

In the battery research field, indirect correlations between electrochemical and physicochemical properties are made using experimental data obtained via ex situ characterizations^3^, i.e., where a specific component of the battery is measured prior to cell assembly or after the electrochemical testing. However, in ex situ measurements, careful harvesting, sampling, preparation and transportation procedures of the specimen are critical to obtain representative, reproducible and reliable data^4^.

To improve the correlation, in situ characterizations^3^ are employed by many researchers in the battery field. These are physicochemical measurements carried out together with the electrochemical experiment, which is paused at a near-equilibrium stage for a specific time to collect the data from the physicochemical measurement. However, these characterizations present limitations because the pause imposed on the cell during the electrochemical experiment may not fully represent the conditions during cycling.

For this reason, *operando* characterizations^3^ with no need to pause the system have gained significant attention in the battery field^5,6^. Indeed, *operando* characterizations could improve understanding of the local properties of both electrochemically active and inactive materials in lab and commercial scale batteries^7^.

“*Operando* characterizations could improve understanding of the local properties of both electrochemically active and inactive materials in lab and commercial scale batteries.”

However, we note that the application of *operando* and in situ terminologies is not always standardized in the broad battery field. Future discussions on this aspect in the research community will be needed to unify the nomenclature used by scientists with different backgrounds.

Here, to showcase the recent developments in the field of *operando* characterizations for electrochemical energy storage systems, we are delighted to unveil a collection of some of the most exciting articles published in *Nature Communications* in the last five years on this topic. In these research articles, by real-time coupling of electrochemical experiments with spectroscopy, microscopy, diffraction and acoustic measurements also using optical Bragg fibers, scientists have improved the understanding of the charge storage processes and electro-chemo-mechanical stability of electrodes and electrolytes (either liquid or solid) for alkali metal-based and multivalent battery systems.
